# Components in downstream health promotions to reduce sugar intake among adults: a systematic review

**DOI:** 10.1186/s12937-023-00884-3

**Published:** 2024-01-17

**Authors:** Syathirah Hanim Azhar Hilmy, Norhasnida Nordin, Mohd Yusmiaidil Putera Mohd Yusof, Tuan Yuswana Tuan Soh, Norashikin Yusof

**Affiliations:** 1 Centre of Population Oral Health and Clinical Prevention, Faculty of Dentistry, Universiti Teknologi MARA (UiTM), Sungai Buloh, Selangor, 47000 Malaysia; 2 Department of Periodontology & Community Oral Health, Faculty of Dentistry, Universiti Sains Islam Malaysia (USIM), Kuala Lumpur, 57000 Malaysia; 3 Centre of Comprehensive Care, Faculty of Dentistry, Universiti Teknologi MARA (UiTM), Sungai Buloh 47000, Selangor, Malaysia; 4 Institute of Pathology, Laboratory and Forensic Medicine (I-PPerForM), Universiti Teknologi MARA (UiTM), Sungai Buloh 47000, Selangor, Malaysia

**Keywords:** Adults, Dietary sugars, Health behaviour, Health promotion, Sugars

## Abstract

Excessive sugar consumption is well documented as a common risk factor for many Non-Communicable Diseases (NCDs). Thus, an adequate intervention description is important to minimise research waste and improve research usability and reproducibility. A systematic review was conducted to identify components in published evidence interventions pertaining to the health promotions on reducing sugar intake among adults. The review was conducted in accordance with the Preferred Reporting Items for Systematic Reviews and Meta-Analyses (PRISMA) statement and used the Mixed Methods Appraisal Tool (MMAT) for quality appraisal. The period for the selected study was from 2000 to 2022, and articles were retrieved from Web of Science (WOS), Medline, Scopus, and PubMed. The target population was adults aged 18 years old and above who underwent intervention to assess the changes in their sugar intake. Data sources and all human epidemiologic studies were included. Out of the 9,333 papers identified, 25 were included. The overall quality of evidence of the studies was considered moderate. Apart from the characteristics of the reviewed studies, components of interventions are including the basis of theoretical or model for the intervention, which majority use Social Cognitive Theory, followed by PRECEDE-PROCEED model, socio-ecological and process-improvement theories and Transtheoretical Model; providers, who are commercial provider, qualified nutritionist, professor of nutrigenomics and nutrigenetics, doctor, dietitian nutritionist, lifestyle coaches, and junior public health nurses; duration of the intervention and follow-up time, varies from as short as one month to as long as 24 months; material provided either softcopy or hardcopy; tailoring approach, based on the individual goals, the process of change, genotype analysis, beliefs, barriers, and sociocultural norms; delivery mechanism either face-to-face or technology-mediated; and tools to measure the sugar consumption outcome mostly used Food Frequency Questionnaire (FFQ), besides 24-h dietary recalls, and food diaries. There are various components in downstream health promotion to reduce sugar intake among adults that can be adapted according to the local health promotion and intervention context. More well-designed interventions using integration components are encouraged in further studies.

**Supplementary Information:**

The online version contains supplementary material available at 10.1186/s12937-023-00884-3.

## Introduction

Modifiable lifestyle-related factors play a large role in an individual’s health. One of them is a nutritional risk factor for non-communicable diseases, such as dietary sugars that have been of considerable high concern and focus among health workers, policymakers, scientists, popular media and the public [1]. The dramatically increased dietary sugar consumption is approximately 171.69 million metric tons in 2019/2020 worldwide and is projected to increase to about 178.84 million metric tons by 2022/2023 [2].

The attention to these excessive empty calories is because it hinders proper growth and development due to its lack of nutrients [3]; ability to decrease the pH in the oral cavity that will promote dental caries [4]; consistent potential to leading to cardiovascular disease (CVD) [5]; and its associated conditions, such as obesity [6], type 2 diabetes mellitus (T2DM) [7], and non-alcoholic fatty liver disease (NAFLD) [8].

It is important to educate and promote the World Health Organisation (WHO) recommendation to limit free sugars intake to less than 10% of the total energy intake for adults and children, observing that a further reduction of 5% would provide additional health benefits [9]. Hence, various health promotion activities aimed at healthy eating habits alone or implemented in conjunction with physical activities are essential for improving quality of life and reducing the prevalence of chronic diseases [10]. Furthermore, because high sugar intake is a common risk factor for many chronic diseases, a Common Risk Factor Approach (CRFA) can be used to create cross-disciplinary health promotion programmes that offer the potential for effectively dealing with a combination of health problems [11]. It is more effective in the long term and has better efficiency in the use of resources [12].

The assessment of interventions is a significant area of study, yet there is a notable deficiency in the quality of intervention descriptions in published works. This lack of comprehensive information hinders other researchers from reproducing or expanding upon research findings. Consequently, the implementation of effective interventions becomes uncertain for clinicians, patients, and decision-makers. Describing an intervention involves more than simply providing a name or listing its ingredients. Crucial aspects such as duration, dosage or intensity, delivery method, essential processes, and monitoring all play a role in its effectiveness and reproducibility, but these details are frequently absent or inadequately explained. In the case of complex interventions, each component requires this level of detail. This systematic review aimed to determine the components of interventions for reducing sugar intake among adults. This review concentrates on the downstream approaches to health promotions based on reducing sugar intake as a common risk factor. Other health settings can, therefore, utilise a combined strategy from this review to be adapted to their field.

## Methods

### Protocol and Registration

A systematic review was performed based on the Preferred Reporting Items for Systematic Review and Meta-Analyses (PRISMA) to ensure methodological and reporting qualified [13]. The registration number for this systematic review is CRD42022323014.

### Research questions

The PICOS principle formulated the research questions (Population, Intervention, Comparator, Outcome, Study) to define the research questions [13]. The review aims to answer the following question: What are the components of downstream health promotion to reduce sugar intake among adults?

“Population” was targeted at adults (aged above 18 years old); “intervention” was focused on health promotion strategies; “comparator” is not applicable in this review; “outcome” was the changes in sugar intake; and “study” was focused on randomised or non-randomised experiment study. Table 1 shows the details of the inclusion and exclusion criteria.

**Table 1:** PICOS and eligibility criteria

Study Characteristics	Inclusion Criteria	Exclusion Criteria
Population (P)	Participants aged 18 years and above. No restrictions on the upper age limit, medical conditions and sex.	Paediatric patients (<18 years), dyad (parents and children).
Intervention (I)	This review will consider public health interventions addressing a reduction in sugar consumption limited to study and intervention on human. Health promotion interventions, such as health education, nutrition education, dietary change strategies, and environmental modifications are done; with content focusing on diet only or on diet and exercise in the settings of schools, workplaces, primary care, the community (cafeterias and restaurants), and supermarkets.	-
Comparison (C)	-	-
Outcome (O)	The change in amount or frequency of sugar. Sugars—other than these intrinsic natural sugars—are classified by WHO as free sugars, which include all monosaccharides and disaccharides added to foods by manufacturers, cook, or consumer, in addition to sugars that are naturally present in honey, syrups, as well as fruit juices and concentrates.	Sugar-related physiological measures behaviour towards sugary change (psychological outcome).
Study design/ publication type (S)	Human studies in randomised controlled trial (RCT) or any others intervention studies (non-RCT), such as quasi-experimental.	Primary medical studies, such as prediction studies, text, and expert opinion papers, editorial, proceeding, abstract, case-reports, clinical practice guidelines, together with secondary review studies such as review articles.
Time frame	1 January 2000 to 3 November 2022	-
Language	English	Non-English

### Search strategy

A literature search was conducted in MEDLINE, PubMed, Scopus, and Web of Science (WOS) databases in the Ovid platform to identify studies related to the research question. The search strategies explore for any similar meaning of related terms and the multiple main keywords for the study.

Identification was aimed to provide more possibilities for searching in selected databases for similar articles for the review using suitable keywords. The review teams have decided and agreed upon the appropriate medical sub-headings (MeSH) main keywords. The result of the keywords searched is demonstrated in Supplementary file 1. Other controlled vocabulary used in indexed journals was considered for developing the strategy.

Furthermore, the search for relevant articles was conducted on selected databases using advanced searching techniques, such as the Boolean operator, phrase searching, truncation, wild card, and field code function separately, or by combining these searching techniques into a full searching string based on the main and enriched keywords, attached in Supplementary file 2. The user guide of the database inquiry also drives the approach of searching.

### Screening of articles for eligibility

Retrieved articles were screened in three phases. In the first phase, any article with titles that did not match the inclusion criteria was excluded. In the second phase, the abstracts of the remaining articles were screened, and any articles that did not meet the inclusion criteria of this study were excluded. In the final phase, full texts of the remaining articles were read and assessed thoroughly to exclude articles that did not meet the inclusion criteria of this study or articles that fulfilled the exclusion criteria. Systematic reviews or meta-analyses were also excluded. Duplicates were removed. All the authors were involved in the selection and the data extraction phase. Any differences in opinions were resolved by discussion between the authors. All data extraction was performed independently using a data collection form to standardise the data collection.

### Assessment of risk of bias in included studies

For the studies included in this review, assessment of risk of bias was conducted by two review authors using the critical appraisal tool, Mixed Methods Appraisal Tool (MMAT) 2018, to appraise the methodological quality of systematic mixed studies reviews, such as randomised controlled trials and non-randomised studies [14]. There were two screening and four methodological quality criteria questions, according to the category of study designs that needed to be answered for each article. All articles were grouped into three distinct quality categories: High (more than three “Yes” answers), Moderate (three “Yes” answers), and Low (less than three “Yes” answers). Most of the articles were ranked as Moderate quality in this review [15]. Outcomes from the MMAT exercise for the 25 papers from the database searches showed that six studies scored 100%, ten scored 75% and nine scored 50% or less. RCT data exhibited a somewhat greater risk of bias (see MMAT summary Table 2).

**Table 2:** Summary of MMAT

Criteria RCT Study	S1	S2	2.1 Randomisation	2.2 Concealment /blinding	2.3 Complete outcomes	2.4 Loss to follow up	Overall score
Miller, 2012 [16]	YES	YES	YES	CAN’T TELL	YES	NO	50%
Hebden, 2014 [17]	YES	YES	YES	YES	NO	NO	50%
Kattelman, 2014 [18]	YES	YES	YES	NO	YES	NO	50%
Nour, 2015 [19]	YES	YES	YES	CAN’T TELL	YES	YES	75%
Hedrick, 2017 [20]	YES	YES	YES	CAN’T TELL	YES	YES	75%
Al Khatib, 2018 [21]	YES	YES	YES	CAN’T TELL	YES	YES	75%
Webel, 2018 [22]	YES	YES	YES	NO	CAN’T TELL	CAN’T TELL	25%
Kaur, 2020 [23]	YES	YES	YES	CAN’T TELL	YES	CAN’T TELL	50%
Manios, 2020 [24]	YES	YES	YES	CAN’T TELL	YES	YES	75%
Islam, 2021 [25]	YES	YES	YES	YES	YES	YES	100%
Rahul, 2021 [26]	YES	YES	YES	YES	YES	YES	100%
Chow, 2021 [27]	YES	YES	YES	YES	YES	NO	75%
Johnstone, 2021 [28]	YES	YES	YES	YES	YES	YES	100%
Mason, 2021 [29]	YES	YES	YES	CAN’T TELL	YES	YES	75%
Average Score							70%
**Criteria** **Non-RCT**	**S1**	**S2**	**3.1 Recruitment**	**3.2 appropriate measures**	**3.3 Comparable groups**	**3.4 Complete outcomes**	**Overall score**
Petrogianni, 2013 [30]	YES	YES	YES	YES	YES	YES	100%
Hietaranta-Luoma, 2014 [31]	YES	YES	YES	YES	YES	YES	100%
Spees, 2016 [32]	YES	YES	YES	YES	CAN’T TELL	NO	50%
Thomson, 2016 [33]	YES	YES	YES	YES	NO	NO	50%
Kendzor, 2017 [34]	YES	YES	YES	YES	CAN’T TELL	YES	75%
Gudzune, 2020 [35]	YES	YES	YES	YES	CAN’T TELL	YES	75%
West, 2020 [36]	YES	YES	YES	YES	CAN’T TELL	CAN’T TELL	50%
Brittain, 2021 [37]	YES	YES	YES	YES	CAN’T TELL	YES	75%
Redmond, 2021 [38]	YES	YES	YES	YES	NO	NO	50%
Goldstein, 2022 [39]	YES	YES	YES	YES	CAN’T TELL	YES	75%
Okube, 2022 [40]	YES	YES	YES	YES	YES	YES	100%
Average Score							73%

### Data extraction and management

Two review authors independently extracted data from the included studies to be presented in a table for comparison. Any disagreements between the two review authors undertaking data extraction were resolved by discussion and the involvement of a third review author.

The following data were extracted from the selected studies: (1) authors’ name and year; (2) country; (3) study design; (4) brief name of intervention; (5) study population; (6) methods to recruit the participants; (7) informed consent; (8) basis of theoretical or model for the intervention; (9) providers; (10) duration of the intervention (11) follow-up; (12) material; (13) tailoring; (14) delivery mechanism; and (15) tools to measure the sugar consumption outcome. The data extraction method was adapted from Hoffman and colleagues, who developed the Template for Intervention Description and Replication (TIDieR) checklist to enhance the intervention details in a systematic review [41].

## Results

### Description of studies

**Fig. 1:**
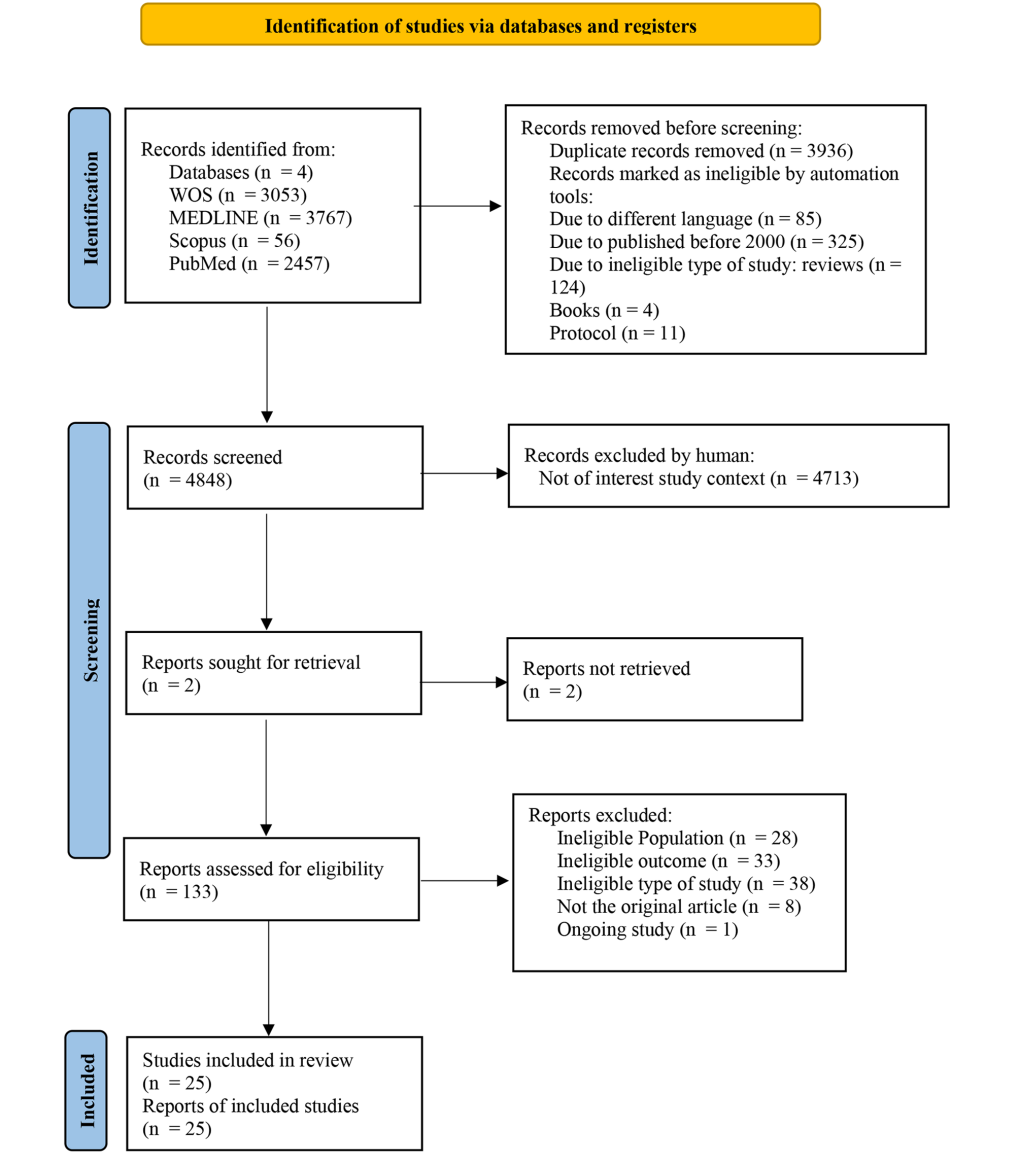
PRISMA 2020 flow diagram for new systematic reviews, which included searches of databases and registers only [152]

The search strategy identified 9,333 articles and approximately 97% of the records were excluded because the articles are not of interest in the study context. Only 134 were selected for full-text screening based on the eligibility assessment and 109 from that were ineligible studies and excluded from this review due to not fulfil the inclusion criteria. At the end of this selected process, twenty-five (25) articles were finally included in this systematic review. A flow chart that summarises the article selection process, and the reasons for article exclusion are shown in Fig. 1. The characteristics of excluded and included studies are reported in Tables 3 and 4 respectively.

**Table 3:** Characteristics of excluded studies

Reason for exclusion	Articles
Ineligible population (n = 28)	[42–70]
Ineligible outcome (n = 33)	[71–104]
Ineligible type of study (n = 38)	[105–143]
Not the original article (n = 8)	[19, 20, 144–150]
Ongoing study (n = 1)	[151]

### Study selection

**Fig. 2:**
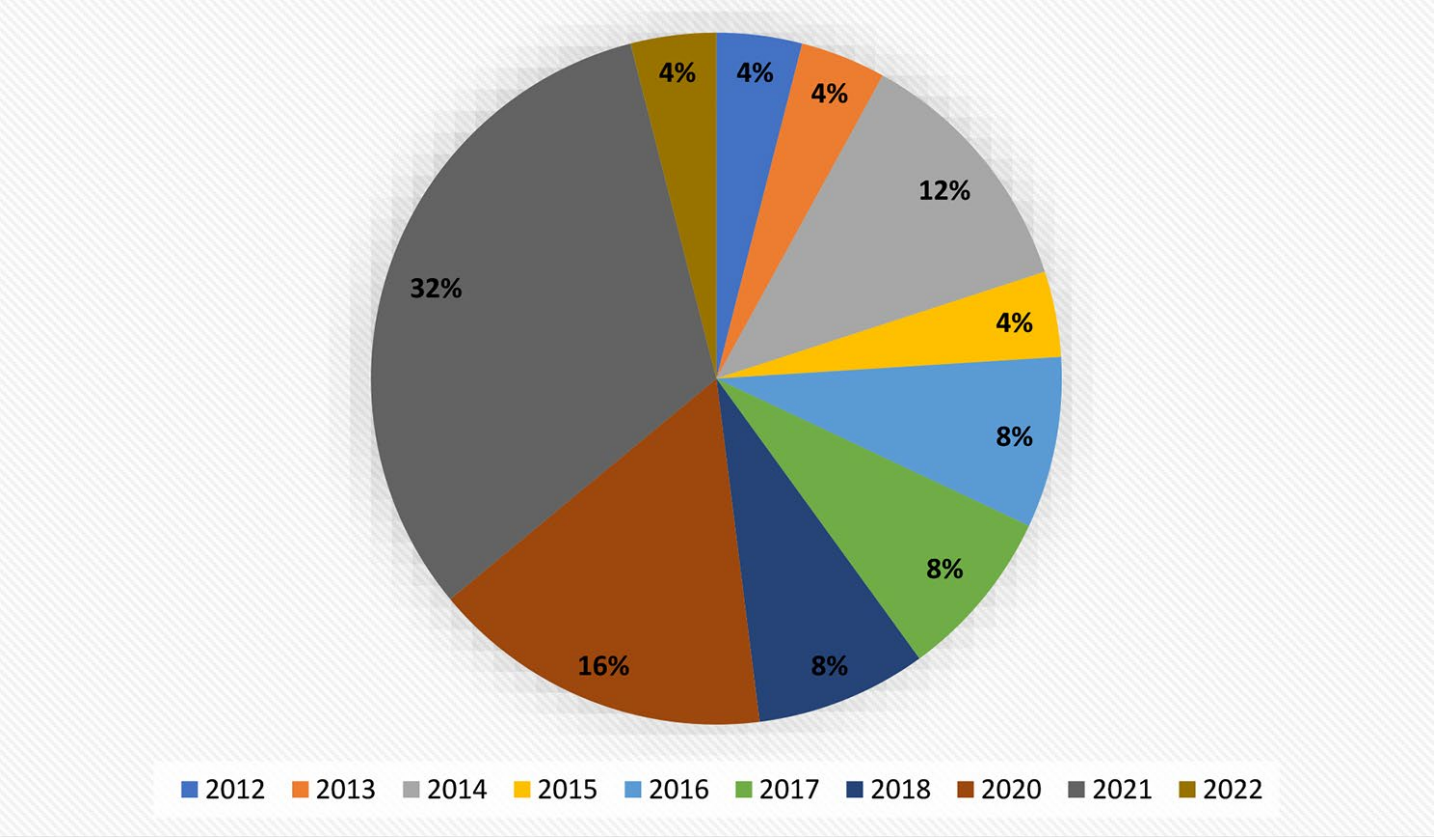
Distribution of included studies by year of publications (percent)

A total of 25 articles were included in this review for further study and analysis, where the majority of the articles were those published in 2021 (Fig. 2). No articles were found prior to 2012. The number of studies originating from each continent are as follows: twelve from the United States of America [16, 18, 20, 22, 27, 29, 32–35, 38, 39]; five from Europe: United Kingdom [21, 28], Greece [24, 30], and Finland [31]; three from Asia: India [23, 26], Bangladesh [25]; three from Australia [17, 19, 36], and a study from New Zealand [37] and Kenya [40]. In addition, a multi-country study was conducted within six countries (Belgium, Bulgaria, Finland, Greece, Hungary, and Spain) [24]. Most of the studies were randomised controlled trials (RCT) [16–29] and others were pre-post intervention studies.

### Sample description

Most of the participants of the study have comorbidities, which focus on type 2 diabetes mellitus (T2DM) patients [16, 25], followed by a sample size (i.e., participants of the study) that was diagnosed with cancer or ongoing cancer treatment [32] and HIV patients [22]. The vulnerable group, such as homeless people [34], is also included in this study. Healthy adults mostly used university or college students as sample [17, 18, 21]. The intervention and control group ratios were different for certain reasons. Most of the included studies were dominated by females, and a study intentionally chose females as participants [28]. Meanwhile, the ethnicity was not well documented; however, the listed ethnics that dominated in the studies are White, Black, Asian [16, 29], African American [22, 33, 35], Caucasian [20], European [37], and Hispanic/Latino [29].

### Allocation and blinding

Methods for participants allocation such as using a random sampling method by Microsoft Excel [23, 31], Stata 14.0 [22], a custom programme [28], random permuted blocks method [26], and other computer-generated programmes [16–18, 21, 25, 29] were recorded in the included studies; while others studies not mentioned specifically how they allocate the participants. Blinding of outcome assessment was not reported in most of the studies. However, a study did mention that participants were aware of the treatment but were blinded to the nature [17], investigators [25], outcome assessors [26], and provider [27] blinded to the participant allocation. Two researchers practiced a double-blind study [28, 40].

### Methods of recruitment and informed consent

The recruitment methods are mostly a combination of the traditional methods, such as brochures, posters, newspapers, and flyers; besides face-to-face methods in the community, registered patients from healthcare facilities; and electronic recruitment by using radio, website, internet advertisement, and social media. A study has recorded an initiative to follow up the mailing invitations with door-knocking attempts [35]. Most studies mentioned the informed consent obtained, but only a few stated clearly whether they were obtained by written [16, 22, 30, 33] or online [18].

### Use of theory or concept

The included studies applied the theory or model for their interventions in health promotion. For example, Health Belief Model [30]; Social Cognitive Theory [30, 32, 36, 38]; Transtheoretical Model [17, 33]; PRECEDE-PROCEED model [18, 23, 24]; HAPA model [24]; Dick and Carey’s Model [18]; Extended Parallel Process model [31]; socio-ecological and process-improvement theories [22, 23, 38]; self-determination theory [27]; social identity theory [35]; cognitive dissonance theory [35]; social influence theory [35]; behaviour change technique (BCT) [37], such as Coventry, Aberdeen & London-Refined (CALO-RE) taxonomy [21]; and communication skills technique, such as MI [29, 32].

### Intervention providers

Certain studies did not mention who are the providers of the intervention specifically. However, Miller [16] mentioned that trained facilitators provided it; Hebden [17] and Islam [25], by a commercial provider that scheduled the sending text message; Hietaranta-Luoma [31] by a qualified nutritionist, professor of nutrigenomics and nutrigenetics, and doctor; Spees [32] and West [36] a trained registered dietitian nutritionist; Gudzune [35] lifestyle coaches; Rahul [26] by junior public health nurses; and Chow [27] and Goldstein [39] by the clinician.

### Duration of the intervention, follow-up and delivery mechanism

The duration varies from as short as one month [21, 28, 34, 37] to as long as 24 months [24] and relatively, three (3) [16, 17, 30] to six (6) months [22, 23, 25, 26, 33, 35, 39]. For the follow-up, the included studies did one-time or repeated follow-ups. The shortest follow-up which is less than one month, day 28 or as close as practical was recorded by Johnstone [28]. Most of the studies do follow-up at 6 months [20, 23, 25, 26, 33, 35]. However, Kattelman [18] did follow up at 10 weeks and 15 months and Okube [40] at 9 and 15 months. These two studies documented the longest follow-up of participants. There are two types of delivery mechanisms: face-to-face (physical) [16, 21–24, 26, 28–40] or technology mediators (online), where most of the interventions combined these two delivery approaches. There are many channels/approaches in technology-mediated communication, such as CD [16], short message services (SMS) text [17, 19, 23, 24], email [17, 18, 23], smartphone applications [17, 19, 27, 39], internet forum [17], telephone call [19, 33], website [19, 23], social networking apps [23], and social media [27].

### Interventions materials, tailoring and assessment tools

There were varieties of materials either softcopy or hardcopy used in the interventions. None of them were the same due to it was created based on the objectives of the studies. Some materials were tailored based on the personal goals [30], the process of change identified [17], genotype analysis [31], health-related beliefs, barriers, and sociocultural norms [19], baseline self-reports [34], email feedback on the participants’ action and coping plans [37]. Overall, the materials are for educational purposes or guidance purposes.

The educational purposes materials are culturally adapted newsletters [24], a lecture on healthy lifestyle and diet [31], Delta Body and Soul cookbook and monthly newsletter featuring nutrition and physical activity [33], Ozharvest’s Everyday (photo-based) Cookbook [36] and Educational posters, newsletter, brochures, flyers, and educational displays [38]. Guidance purposes materials, for example, the “SMART Eating” kit– kitchen calendar, dining table mat, and measuring spoons [23], a diet tracking app and access to private Facebook group [27] and the help sheet which details a range of barriers and potential solutions [37].

The assessment tools for sugar reduction were mostly questionnaires. The Food Frequency Questionnaire (FFQ) was the predominant tool to measure sugar consumption outcomes with various adaptations; FFQ [24, 26, 27, 36, 38, 40], the 158-item Delta FFQ [33], the FFQ adapted for sugar consumption in the local context [37], a single-item question added in a FFQ known as the Dietary Questionnaire for Epidemiological Studies version 2 (DQESv2) to determine consumption of sugar-sweetened beverages [19], the valid 110-item Block 2005 FFQ (nutrition quest) [16] and Indian Migration Study FFQ [25]. Besides, food diaries [21, 28] and 24-hour dietary recalls [20, 22, 30, 34, 39] are the other tools that were mostly used.

Most of the included studies were multi-component interventions that normally incorporate physical activity and the dietary components, including changes in sugar intake, become the primary or secondary outcome. The details of the components of interventions are attached in Supplementary file 3.

**Table 4:** Characteristics of included studies

Author (Year) Country/ies	Study Design	Brief Name of Intervention	Sample Description (Gender; Mean Age; Group Allocation)	Method(s) of Recruitment	Informed Consent
Miller (2012) The United States of America [16]	RCT 2-arm.	MB-EAT for Diabetes (MB-EAT-D) Mindfulness-based Intervention.	52 T2DM patients (38.5% male; 53.95 years; IG = 27/CG = 25).	Through local medical practices, the university’s newswire, radio, and internet advertisements, and community flyers.	Written informed consent.
Petrogianni (2013) Greece [30]	Pre-post study.	Multicomponent intervention diet and physical activity intervention.	108 hypercholesterolemic adults (53.5% male; 48.7 years; IG = 77/CG = 31).	NM.	Written informed consent.
Hebden (2014) Australia [17]	RCT 2-arm.	mHealth technologies for weight management in young adults.	51 university students and staff (19.6% male; 22.85 years; IG = 26/CG = 25).	Advertisements posted around the university campus and published in staff and student newsletters.	NM.
Hietaranta-Luoma (2014) Finland [31]	Pre-post study.	Genotype-based nutrition and health information intervention.	107 healthy adults (30.8% male; 47.0 years; IG = 61/CG = 61).	NM.	NM.
Kattelman (2014) The United States of America [18]	RCT 2-arm.	Young Adults Eating and Active for Health (YEAH) in a college setting.	1639 college students (33.0% male; 19.3 years; IG = 824/CG = 815).	Face-to-face methods, e.g., in-class and residential life housing meetings, and e-mails, letters, and flyers were posted on participating campuses.	Online informed consent.
Nour (2015) Australia [19]	RCT 2-arm.	TXT2BFiT, a mobile phone-based healthy lifestyle.	250 young adults (35.6% male; 27.7 years).	Recruited from the Greater Sydney area in New South Wales, Australia.	NM.
Spees (2016) The United States of America [32]	Pre-post study.	Growing Hope Multifaceted intervention delivered within a garden setting.	22 cancer survivors (22.7% male; 59 years).	Study brochures were distributed at the cancer hospital, its associated oncology clinics, and its affiliated community-based cancer survivor outreach programme.	Obtained informed consent.
Thomson (2016) The United States of America [33]	Pre-post study.	Delta Body and Soul III lifestyle intervention.	409 participants (28.11%male; 47.15 years; IG = 287/CG = 122).	Via mailed study invitation letters, followed by telephone contact.	Written informed consent.
Kendzor (2017) The United States of America [34]	Pre-post study	Diet and physical activity intervention for homeless adults.	32 shelter residents (75.0% male; 48.38 years; IG = 17/CG = 15).	Weekly orientation meetings, flyers, and word of mouth at the transitional homeless shelter.	Obtained before screening for eligibility from interested individuals.
Hedrick (2017) The United States of America [20]	RCT 2-arm.	Talking Health.	292 participants (19% male; 42 years; IG = 149/CG = 143).	Active recruitment at health department, day care centres, festivals, and others. Passive recruitments from targeted mailings, flyers, radio, and others.	Written informed consent.
Al Khatib (2018) The United Kingdom [21]	RCT 2-arm.	Sleep Lengthening and Metabolic health, Body composition, Energy balance and cardiovascular Risk (SLuMBER).	42 habitually short sleepers (16% male; 24 years; IG = 21/CG = 21).	Internal circular e-mails among university staff and students, as well as social media advertisements and recruitment posters that were publicly available.	Written informed consent.
Webel (2018) The United States of America [22]	RCT 2-arm.	SystemCHANGE is an innovative self-management.	106 HIV + adults (65% male; 53 years; IG = 51/CG = 50).	Via letters sent to an HIV research registry and flyers posted in HIV care organisations.	Written informed consent.
Gudzune (2020) The United States of America [35]	Pre-post study.	Peer outreach approach to reduce added sugar intake by promoting sugar-sweetened beverages (SSB) reduction.	34 participants (20.6% male; 45.7 years). 17 residents/17 network members).	Mailing invitations to all residences and perform follow-ups with these mailings with door-knocking attempts.	NM.
Kaur (2020) India [23]	RCT 2-arm.	SMART Eating website.	732 participants (23.9% male; 52.7 years; IG = 366/CG = 366).	Purposely chosen.	Written and verbal consent after briefing.
West (2020) Australia [36]	Pre-post Study.	OzHarvest’s NEST Programme promoting food security and food literacy.	21 participants (42.9% male; age from 18 to 74 years; no control group).	Recruit by organisations hosting.	Obtained informed consent.
Manios (2020) Belgium, Bulgaria, Finland, Greece, Hungary and Spain [24]	RCT 2-arm.	Feel4Diabetes.	2756 patients at high risk developing T2DM (33.6% male; 40.9 years; IG = 1526/CG = 1230).	Based on a standardised, multi-stage sampling procedure.	Written informed consent.
Brittain (2021) New Zealand [37]	Pre-post study.	Sugar Habit Hacker.	128 adults (12.5% male; 40.46 years; no control group).	A combination of flyers and online advertisements (social media, university, and health promotion websites).	Obtained informed consent.
Islam (2021) Bangladesh [25]	RCT 2-arm.	Text messaging intervention.	236 patients with T2DM (45.8% male; 48.1 years; IG = 106/CG = 94).	From a tertiary hospital.	Written informed consent.
Rahul (2021) India [26]	RCT 2-arm.	Primary care and public health nurses training.	132 participants (24.2% male; 62.7 years; IG = 72/CG = 60)	Recruited through local public service commissions.	Obtained informed consent.
Redmond (2021) The United States of America [38]	Pre-post study.	Obesity Prevention and Evaluation of InterVention Effectiveness in NaTive North Americans (OPREVENT).	299 participants (29.2% male; 44.5 years; IG = 182/CG = 117).	Recruited from each community.	Written informed consent.
Chow (2021) The United States of America [27]	RCT 2-arm.	Individualised goalsetting on diet and physical activity.	41 cancer survivors (51.2% male; 45.1 years median age; IG = 24/CG = 17).	From the designated comprehensive cancer centre.	Obtained informed consent.
Johnstone (2021) The United Kingdom [28]	RCT 2-arm.	Galacto-oligosaccharides (GOS) intervention, dietary changes via prebiotic supplement.	64 healthy young adult female volunteers (0% male; 20.02 years; IG = 23/CG = 23).	Via posters and online advertisements.	Written informed consent.
Mason (2021) The United States of America [29]	RCT 2-arm.	SSB sales ban and Motivational Interviewing (MI).	214 participants. (42.1% male of IG; 41.2 (11.0) years mean age of IG; IG = 109/CG = 105).	From a pool of university employees (staff and faculty). who responded to an initial employee online survey on SSB consumption.	NM.
Goldstein (2021) The United States of America [39]	Pre-post study.	Dietary lapses.	32 adults with overweight/obesity (68.8% male; 54.5 years; no control group).	Via advertisements in local newspapers, the research centre’s website, email newsletters through a hospital and by physician referrals.	Obtained informed consent.
Okube (2022) Kenya [40]	Pre-post study.	Lifestyle intervention targeting common behavioural risk factors metabolic syndrome and cardiovascular disease.	294 participants (38.0% male; 18–64 years old; IG: 156/CG: 138).	Who visited the hospital as outpatients, as well as those who escorted clients as relatives or friends.	Obtained informed consent.

NM = Not mentioned

## Discussion

This paper can be a good starting point for researchers to understand the various interventions and review existing work related to proposed research questions. In this section, a discussion of the analysed publications was presented to show how the retrieved publications answered the proposed research questions. The interventions’ components are crucial in contributing to the success, where the final objective is to reduce and prevent non-communicable diseases caused by excessive sugar consumption.

In this review, the overall quality of evidence of the included studies was considered moderate to high quality, varying in the components of the interventions from the participants’ description, allocation, and blinding, intervention providers, duration, material, underpinned theory, tailoring, mode of delivery, and assessment tools.

Most of the reviewed studies were on the vulnerable adult population and adults with comorbidities. These groups share common characteristics in that they are at risk of diseases, and face barriers to maintaining their health and accessing health facilities. It must be remembered that these people also find themselves at the lower end of the social gradient because of political and social drivers [12]. Hence, in designing interventions to promote better health, it is important to be aware of the context, settings, and circumstances in which some individuals and groups live. Studies were included conducted mostly in developed countries mostly in western countries. Hence, the applicability of intervention and findings to low- and middle-income countries and across different cultures remain unknown.

The usual limitation reported in the reviewed studies was the small sample size, and the lack of a control group may have limited power to detect statistically significant differences. However, the size was appropriate for a feasibility pilot study. Those researchers also should consider a Hawthorne effect, whereby the mere presence of the intervention, not the intervention itself, is associated with favourable changes in outcome measures. In addition, biases that might be associated with drop-out rates, although minimal, may have resulted in an overestimation of the effect of the intervention. The predominantly participants by race, gender, or certain age group also were recognised most in the reviewed papers that limited the generalisability to other sociodemographic groups. In addition, internal recruitments limit the external validity such as the study by Hebden [17]. Therefore, the next intervention should target other groups but must be culturally tailored to be more acceptable to participants from different racial/ethnic backgrounds [28].

The lack of ability to blind participants to the allocation of the intervention group may have introduced confounding effects in the control condition by indirectly stimulating an interest in the primary outcome of the study [21]. There exists a possibility of some degree of contamination. This could be minimised by informing the intervention group health workers not to discuss the information in the training module with their colleagues throughout the trial period [26]. In the future, blinding assessors may be considered to minimise sources of bias.

Other professionals such as teachers, managers, or those working in the fitness industry have an important role in disseminating health messages. In 2003, the WHO recommended that the training of all health professionals, including physicians, nurses, dentists, and nutritionists, should include diet advice in their delivery services [153]. Therefore, it was observed that a few studies used professionals with relevant qualifications in the field of nutrition and dietetics provided the interventions to ensure the most effective dietary variables showed clinically relevant results [32, 36, 154]. Moreover, in psychology, counsellors use empathy and other techniques to create an atmosphere to help patients to explore the discrepancies between their goals and their current behaviour. These findings showed that various occupations can contribute to promoting healthy lifestyles and are not limited to clinicians only.

The range of intervention’s duration raises questions if any dietary changes observed in the shorter follow-up period were sustainable longer term and sufficient to bring about the general benefits of reducing sugar intake. A short-term follow-up as short as a month intervention [21] indicated the intervention had a positive effect in the short term but may have been inadequate to allow for adaptation. On the other hand, in a study by Hietaranta-Luoma [31], the short- and long-term follow-up were measured with the justification that the first 6 months were deemed the active period. In comparison, the following 6 months were a “silent” period designed to stimulate life. Considering the sustainability of the intervention, the 6 months evaluation period is relatively short, and commonly, the enthusiasm for lifestyle changes decreases during the interventions [155]. Further, the effect tended to tail away during the silent period. Even a very strong motivator, may not be powerful enough to stimulate a persistent lifestyle change in a short-term intervention resulting from the present study [31]. Therefore, the intervention needs further follow-up assessments [26] to determine if sugar consumption remains low in the time following the intervention besides being more likely to lead to the adoption of a longer-term lifestyle change. The 6 months is a common benchmark, followed by a less intensive “maintenance” phase to help sustain any intervention effects [27]. Furthermore, assessments might be conducted repeatedly over the course of 6 months to observe participants’ experiences across multiple phases of the intervention [39].

The findings from Hebden and colleagues [17] suggested that the booklet and brief counselling session may be sufficient for young adults to make positive changes to their diet. However, this may only be generalisable to the recruited highly motivated and well-educated sample. In another reviewed paper, the intervention’s materials were rated very useful, and participants were mostly satisfied with the programme [37]. Other reviewed study among older participants reported they appreciated printed materials [23]. The interventionist should consider the involvement of materials advice in correspondence to the current state of nutrition research, which is an evidence-based method despite its high variations within countries and between professions. Therefore, course accreditation of a defined core curriculum is needed in the area of nutrition health education, including information on sugars and health, for all health professionals, educators, caregivers, and other relevant professions, to ensure consistency in providing accurate messages across professions [156].

But, the offline type materials have drawbacks if the participants were living under one roof as mentioned in Kendzor’s [34] because the control participants might be able to access the intervention material. For this reason, an online newsletter should be considered in future research to minimise the bias of the intervention. However, brief e-mail nudges may not be sufficiently powerful to maintain behaviour change [157], even though IT approaches either electronic or mobile (e- or m-commerce) demonstrate the feasibility and acceptability among the urban population and can minimise the limitations of resources and geographical distances, especially for low-income strata populations. Nevertheless, the common barrier in smartphone applications was the slow operating speed of the application itself. In addition, low computer literacy was evident in a subset of older participants [23, 158] and the password protection of the website or application could be a drawback if participants forget the password, making it difficult for the users to log in and leading to low engagement among participants. These participants preferred face-to-face or telephonic contact and had little interest in navigating the website. Besides that, using text messaging intervention on its own may serve populations where smartphone access is limited, such as in rural areas of Bangladesh and lower socioeconomic areas [154].

The delivery by SMS text messages such as that applied in Hebden’s study [17] indicates that this method can potentially reduce SSB intake. However, it may not be beneficial for reducing total energy intake. This result has a similar finding as seen in a systematic review [159] that might be because SMS text messages require the cost or time constraints that lead to limited engagement when they do not reply to all sent messages [17]. Another study [25] found that a text messaging programme in people with T2DM did not significantly improve dietary intake and a study (44) reported the generalisation of the text messages used in the intervention may have hindered the ability of participants to reach their full potential in improving dietary habits.

Suppose text messages contained more specific information about what comprises a healthy diet and how to achieve it if it were personalised to the intervention. In that case, it will enhance the ability of the participants to change their behaviour.

However, knowledge alone may not work and only give a limited impact, there is a need to facilitate the change process from the inner strength of the individuals. It is aligned with the study by Roe in 1997 mentioned interventions should be developed from behavioural theory and incorporate well-defined goals [160]. By understanding all relevant background information, a good rapport could be established between providers and participants, further, this personal contact might be important in motivating and monitoring an individual’s change. In this review, many interventions underpinned by psychological theory such as in the NEST programme underpinned by Social Cognitive Theory, and this programme aim to build self-efficacy in its participants that have been shown to improve an individual’s capability in utilisation dimensions of the food security [36]. Another example is the effectiveness of the “nutrition and lifestyle counselling” component of the programme with respect to increasing the self-efficacy of the intervention participants to comply with the given health behaviour instructions in the intervention group [30]. Initially, self-efficacy can be enhanced through reminders.

On the other hand, there are many factors that influence readiness to change, as seen in a study framed by the Transtheoretical Model (TTM) [161]. They chose not to engage because they did not perceive that change as important, or feel they have adequate support, or are uncertain about the impact of such behaviours on their health. Another study found that some participants were still in denial by declining help from the motivational interviewing (MI) coach and felt they did not have enough time or did not need coaching to achieve their goals [32]. Therefore, it would be much easier if the providers could assess the readiness for behaviour change at baseline during the screening process as the intervention can be more focused on the content, intensity, and duration needed [162].

The health promotion programmes must also be tailored to fit patients’ priorities and goals. Besides that, the tailoring intervention according to participants’ age, location, and socioeconomic status should be adjusted [25] based on the therapeutic alliance, cost-effectiveness, and sustainability over the medium and long term. By empowering patients with the necessary knowledge and skills to attain a positive mental attitude and change their locus of control from an external to an internal one [163] together with accessibility in real-time when needed [164], the interventions’ effectiveness especially for a narrower population may be increased. Study by Hietaranta-Luoma et al. (2014) provides personal genetic information, in combination with a personal health message, had slight, favourable effects on dietary and lifestyle choices. It is in line to give an encouraging message that personal lifestyle choices can impact an individual’s health and risk factors [31].

The most reviewed paper used MI in tailoring interventions. This collaborative and patient-centred counselling approach aims to elicit behaviour change by identifying strategies for behaviour change that are motivational (e.g., realising, examining the pros and cons of change, and seeking information and knowledge) [165]. It focuses on finding and resolving the ambivalence, improving patients’ perception of the importance of behaviour change, and supporting them to make the change while providing a structural framework with guiding principles [166] that can be easily utilised by a variety of local healthcare providers that understand the context of the local residents, which makes it adaptable for different culture and clinical settings [167]. MI appears to be a promising approach for changing individual behaviour in many health outcomes including improving healthy eating [168] and can be sustained at 3 and 6 months after MI intervention [169]. No statistically significant differences were found between individual and group delivery modes [170]. However, face-to-face counselling sessions were inconvenient due to a lack of time to attend the session [24]. As an alternative, a remote MI can be an alternative to the physical meetings in providing additional support [171] to participants.

Although the content and modes of delivery vary enormously, a supportive environment such as in schools and neighbourhoods [24] should be created to get a promising result in reducing sugar intake. Support from friends and family was reported as an enabler for sustaining food security or protecting from the worst aspects of food insecurity [172]. In addition, there is sound evidence that engagement from the group, social and peer support [161] can increase the effectiveness of dietary interventions, where, as part of the goal-setting activity the NEST programme encourages participants to reflect on whom they could share the information with [36]. A clinically meaningful and statistically significant decrease in added sugar intake by using the participant’s social network member’s approach. Hence, it demonstrates the promising acceptability, implementation, and efficacy of the social support involvement in the intervention. Therefore, future interventions can assess the effect of engaging social support in supporting participants’ change behaviour.

The variations of delivery mechanisms in the interventions to reduce sugar intake are mostly divided into face-to-face, technology-mediated, or a combination of both mechanisms. This component should be considered as one of the factors to ensure the intervention succeeds. It is because every mode of delivery has its benefit and drawbacks. Furthermore, a comprehensive comparison could be conducted to understand the influence of this component. The effectiveness of the intervention’s mode of delivery should be tested in a controlled setting and needs further exploration through implementation research before its potential scale-up.

Identification of appropriate dietary outcome measures will be a challenge; for it will probably require more than one type of measure to be used (e.g., frequency as well as the amount of sugar consumption). In this review, overall, the assessment of the change in sugar outcome in included studies was not broadly measured. Most of the included studies in this review only measured the quantity of sugar intake by using the questionnaire tool or diet diary. Surveying the intake of foods and drinks such as food frequency questionnaires and 24-hour recalls are the common methods for assessing the dietary intake of a population. The 24-hour recall is considered to offer a favourable balance of cost-effective, scalable, acceptable accuracy of dietary intake and impose a low subject burden to reduce the likelihood of participant attrition and misreporting because of reactivity bias (e.g., changes in respondents’ eating behaviour in response to the act of recording) [173]. However, recalling intake even for the previous day is a challenging task for some individuals. For example, people with reduced cognitive and memory abilities (e.g., fading memory and reduced attention span) [174] can contribute significantly to underreporting of dietary intake. Furthermore, the serving size that a respondent remembers that they ate, the portion size consumed in reality and specific details of recipes used for cooking the reported foods can easily misreport its ingredients especially, if the meal was not cooked by the respondent [175]. Recently, 24-hour diet recalls were adopted in a web-based assessment, where thousands of self-administered manners can record and submit their dietary recalls remotely. However, it has its limitations, including errors related to human memory by allowing the use of shorter retention intervals in certain studies that could potentially improve the accuracy of dietary assessment [176].

Ideally, a combination of dietary outcome measures including amount, frequency, choices, purchases, biochemical, anthropometry, cognitive, behavioural measures, and psychological measurements would give better predictors of reducing sugar intake and a comprehensive result of the conducted intervention. Future studies may need to use a greater range and complexity of dietary behaviour outcome measures.

Most of the included studies in this review were multifaceted interventions. This complex intervention with its properties such as the number of components involved; the range of behaviours targeted; expertise and skills required by those delivering and receiving the intervention; the number of groups, settings, or levels targeted; or the permitted level of flexibility of the intervention or its components [177] resulted in difficulty to differentiate the “active ingredients” and how they relate to each other or the greater the likelihood that one is dealing with [178]. Where complex interventions are involved, the possibility of a synergistic effect of various components should be examined [179]. In contrast, biomedical interventions are precisely specified (e.g., the pharmacological “ingredients“ of prescribed drugs, their dose and frequency of administration) [180] as seen in a study by Johnstone [28]. Hence, any exploration of individual behaviour change needs to consider the influence of the broader factors operating at a macro level [12]. Given that behaviour change is a difficult and complex process which sometimes are outside of the control of the individual, further work is needed to determine the sustainability of intervention effect along with exploratory research on understanding barriers to sustainability.

Intervention studies on reducing sugar intake among adults have been conducted across the globe among diverse populations and setting as excessive sugar consumption is well documented as a common risk factor for many NCDs. The involvement of sugar in oral and systemic diseases is crucial. Therefore, adapting the Common Risk Factor Approach (CRFA) as a holistic perspective in targeting the individual approach in a downstream preventive application is important, but it must be culturally competent, considering patients’ beliefs and perceptions. Moreover, future studies should apply a randomised controlled trial design to determine whether the specific intervention is more effective than no treatment. It would also be useful to test the intervention with and without coaching to determine the relative contribution of each intervention component.

### Study limitation

Our systematic review has limitations. Firstly, the review of the interventions’ feasibility, acceptability, and rate of retention cannot be done in a single article, and it will be continued in another article to provide a further understanding of this whole systematic review. Next, despite conducting a systematic review, it is also encouraged to look objectively or perform a meta-analysis. However, the scope of this review was broad, and the collected data were heterogenous, so it was impossible to develop a meta-analysis with these data. Lastly, it is expected that the article will highlight quality variations if the checking is based on different quality assessment tools. However, Shaffril and Samah [181] emphasized that quality assessment is not solely intended to find the perfect article but rather to find articles that fit the purpose of the review. Therefore, the researcher would like to recommend that the scope for further study be narrowed so that a comprehensive review and meta-analysis can be done.

## Conclusion

This review analysed multi-components of interventions to reduce sugar intake among adults, including vulnerable groups with the most used Social Cognitive Theory; a variation in provider types from non-health practitioners to health professors; duration of the intervention from as short as one month to as long as 24 months; with follow-up time as close as practical time to as long as 15 months, either one time or repeated follow-ups; delivery mechanism by using face-to-face or technology-mediated; softcopy or hardcopy with educational or guidance purposes material with some interventions are using tailoring approach and FFQ as a tool to measure the sugar consumption outcome were mostly used across interventions. This review provides useful insights to adapt components based on different health settings’ practicability and affordability. More well-designed interventions using integration components are encouraged in further studies.

### Electronic supplementary material

Below is the link to the electronic supplementary material.

Supplementary Material 1

Supplementary Material 2

Supplementary Material 3

Supplementary Material 4

## Data Availability

Not applicable.
