# Supplementary material for: Components in downstream health promotions to reduce sugar intake among adults: a systematic review
Source: Nutr J. 2024 Jan 17;23:11. doi: 10.1186/s12937-023-00884-3 (PMC10792802; doi:10.1186/s12937-023-00884-3)
Supplement: Supplementary file 1 — Supplementary Material 1 [file 12937_2023_884_MOESM1_ESM.docx]

Supplementary file 2 of Search strategies for databases

| **No** | **Search String: Web Of Science (WOS)** | **Results** |
| --- | --- | --- |
| 1 | TS=(*promot* OR *guide* OR *advise* OR *advice* OR *educat* OR *motivat* OR *counsel* OR *info* OR *analysis* OR *train* OR *demonstrat* OR *instruct* OR *behavio* OR *modif* OR *attitude* OR *service*) | 27,296,991 |
| 2 | **TS=(*health* OR *oral* OR *dental* OR *nutri* OR *diet*)** | 8,148,999 |
| 3 | **#1 AND #2** | 4,736,339 |
| 4 | **TS=( *sugar* OR *ssb* OR 'free sugar' OR 'added sugar' OR 'sugar-sweetened beverages' OR 'cariogenic diet' OR 'dietary sucrose' OR 'dietary sugars' OR 'high fructose corn syrup' OR 'dietary carbohydrates' OR 'carbonated beverages')** | 443,067 |
| 5 | **TS=(knowledge OR attitude OR practice OR behav* OR capable* OR opportunity OR motivation*)** | 9,190,427 |
| 6 | **#4 AND #5** | 57,573 |
| 8 | **#6 AND #3** | 16,834 |
| 9 | **ALL=(adult*)** | 1,985,793 |
| 10 | **#8 AND #9** | 3053 |
| 11 | **#10** and **1999** or **1998** or **1997** or **1996** or **1995** or **1994** or **1993** or **1992** or **1991** or **1980** or **1971** (Exclude – Publication Years) | 2909 |
| 12 | **#10** and **1999** or **1998** or **1997** or **1996** or **1995** or **1994** or **1993** or **1992** or **1991** or **1980** or **1971** (Exclude – Publication Years) and **Editorial Material** or **Book Chapters** or **Meeting Abstract** or **Reprint** or **Correction** (Exclude – Document Types) | 2891 |
| 13 | **#9** and **1999** or **1998** or **1997** or **1996** or **1995** or **1994** or **1993** or **1992** or **1991** or **1980** or **1971** (Exclude – Publication Years) and **Editorial Material** or **Book Chapters** or **Meeting Abstract** or **Reprint** or **Correction** (Exclude – Document Types) and **French** or **German** or **Portuguese** or **Spanish** or **Greek** or **Icelandic** or **Korean** or **Czech** or **Hungarian** or **Turkish** or **Indonesian** or **Italian** or **Japanese** | 2787 |

| **No** | **Search String: MEDLINE** | **Result** |
| --- | --- | --- |
| 1 | (promot* or guide* or advise* or advice* or educat* or motivat* or counsel* or info* or analysis* or train* or demonstrat* or instruct* or behavio* or modif* or attitude* or service*).mp. [mp=ti, ab, tx, ct, bt, ot, nm, hw, fx, kf, ox, px, rx, ui, sy] | 24,626,268 |
| 2 | (health* or oral* or dental* or nutri* or diet*).mp. [mp=ti, ab, tx, ct, bt, ot, nm, hw, fx, kf, ox, px, rx, ui, sy] | 12,667,388 |
| 3 | 1 and 2 | 9,500,158 |
| 4 | (sugar* or ssb* or 'free sugar' or 'added sugar' or 'sugar-sweetened beverages' or 'cariogenic diet' or 'dietary sucrose' or 'dietary sugars' or 'high fructose corn syrup' or 'dietary carbohydrates' or 'carbonated beverages').mp. [mp=ti, ab, tx, ct, bt, ot, nm, hw, fx, kf, ox, px, rx, ui, sy] | 393,357 |
| 5 | 3 and 4 | 193,687 |
| 6 | adult*.mp. [mp=ti, ab, tx, ct, sh, bt, ot, nm, hw, fx, kf, ox, px, rx, an, ui, sy] | 8,680,414 |
| 7 | ((promot* or guide* or advise* or advice* or educat* or motivat* or counsel* or info* or analysis* or train* or demonstrat* or instruct* or behavio* or modif* or attitude* or service*) and (health* or oral* or dental* or nutri* or diet*) and (sugar* or ssb* or 'free sugar' or 'added sugar' or 'sugar-sweetened beverages' or 'cariogenic diet' or 'dietary sucrose' or 'dietary sugars' or 'high fructose corn syrup' or 'dietary carbohydrates' or 'carbonated beverages') and adult*).ab. | 3767 |

| **No** | **Search String: SCOPUS** | **Results** |
| --- | --- | --- |
| 1 | TITLE-ABS-KEY ( *promot* OR *guide* OR *advise* OR *advice* OR *educat* OR *motivat* OR *counsel*OR *info* OR *analysis* OR *train* OR *demonstrat* OR *instruct* OR *behavio* OR *modif* OR*attitude* OR *service* ) | 41,674,559 |
| 2 | TITLE-ABS-KEY ( *health* OR *oral* OR *dental* OR *nutri* OR *diet* ) | 12,811,778 |
| 3 | ( TITLE-ABS-KEY ( *promot* OR *guide* OR *advise* OR *advice* OR *educat* OR *motivat* OR *counsel*OR *info* OR *analysis* OR *train* OR *demonstrat* OR *instruct* OR *behavio* OR *modif* OR*attitude* OR *service* ) ) AND ( TITLE-ABS-KEY ( *health* OR *oral* OR *dental* OR *nutri* OR *diet* ) ) | 8,312,195 |
| 4 | TITLE-ABS-KEY ( *sugar* OR *ssb* OR 'free AND sugar' OR 'added AND sugar' OR 'sugar-sweetened AND beverages'OR 'cariogenic AND diet' OR 'dietary AND sucrose' OR 'dietary AND sugars' OR 'high AND fructose AND corn ANDsyrup' OR 'dietary AND carbohydrates' OR 'carbonated AND beverages' ) | 80 |
| 5 | ( ( TITLE-ABS-KEY ( *promot* OR *guide* OR *advise* OR *advice* OR *educat* OR *motivat* OR *counsel* OR*info* OR *analysis* OR *train* OR *demonstrat* OR *instruct* OR *behavio* OR *modif* OR *attitude* OR*service* ) ) AND ( TITLE-ABS-KEY ( *health* OR *oral* OR *dental* OR *nutri* OR *diet* ) ) ) AND ( TITLE-ABS-KEY( *sugar* OR *ssb* OR 'free AND sugar' OR 'added AND sugar' OR 'sugar-sweetened AND beverages' OR 'cariogenicAND diet' OR 'dietary AND sucrose' OR 'dietary AND sugars' OR 'high AND fructose AND corn AND syrup' OR 'dietaryAND carbohydrates' OR 'carbonated AND beverages' ) ) | 56 |

| **No** | **Search String: PUBMED** | **Result** |
| --- | --- | --- |
| 1 | *promot*[Title/Abstract] OR *guide*[Title/Abstract] OR *advise*[Title/Abstract] OR *advice*[Title/Abstract] OR *educat*[Title/Abstract] OR *motivat*[Title/Abstract] OR *counsel*[Title/Abstract] OR *info*[Title/Abstract] OR *analysis*[Title/Abstract] OR *train*[Title/Abstract] OR *demonstrat*[Title/Abstract] OR *instruct*[Title/Abstract] OR *behavio*[Title/Abstract] OR *modif*[Title/Abstract] OR *attitude*[Title/Abstract] OR *service*[Title/Abstract] | 12,399,682 |
| 2 | *health*[Title/Abstract] OR *oral*[Title/Abstract] OR *dental*[Title/Abstract] OR *nutri*[Title/Abstract] OR *diet*[Title/Abstract] | 5,012,571 |
| 3 | (*promot*[Title/Abstract] OR *guide*[Title/Abstract] OR *advise*[Title/Abstract] OR *advice*[Title/Abstract] OR *educat*[Title/Abstract] OR *motivat*[Title/Abstract] OR *counsel*[Title/Abstract] OR *info*[Title/Abstract] OR *analysis*[Title/Abstract] OR *train*[Title/Abstract] OR *demonstrat*[Title/Abstract] OR *instruct*[Title/Abstract] OR *behavio*[Title/Abstract] OR *modif*[Title/Abstract] OR *attitude*[Title/Abstract] OR *service*[Title/Abstract]) AND (*health*[Title/Abstract] OR *oral*[Title/Abstract] OR *dental*[Title/Abstract] OR *nutri*[Title/Abstract] OR *diet*[Title/Abstract]) | 2,684,489 |
| 4 | *sugar*[Title/Abstract] OR *ssb*[Title/Abstract] OR 'free sugar'[Title/Abstract] OR 'added sugar'[Title/Abstract] OR 'sugar-sweetened beverages'[Title/Abstract] OR 'cariogenic diet'[Title/Abstract] OR 'dietary sucrose'[Title/Abstract] OR 'dietary sugars'[Title/Abstract] OR 'high fructose corn syrup'[Title/Abstract] OR 'dietary carbohydrates'[Title/Abstract] OR 'carbonated beverages'[Title/Abstract] | 153,379 |
| 5 | ((*promot*[Title/Abstract] OR *guide*[Title/Abstract] OR *advise*[Title/Abstract] OR *advice*[Title/Abstract] OR *educat*[Title/Abstract] OR *motivat*[Title/Abstract] OR *counsel*[Title/Abstract] OR *info*[Title/Abstract] OR *analysis*[Title/Abstract] OR *train*[Title/Abstract] OR *demonstrat*[Title/Abstract] OR *instruct*[Title/Abstract] OR *behavio*[Title/Abstract] OR *modif*[Title/Abstract] OR *attitude*[Title/Abstract] OR *service*[Title/Abstract]) AND (*health*[Title/Abstract] OR *oral*[Title/Abstract] OR *dental*[Title/Abstract] OR *nutri*[Title/Abstract] OR *diet*[Title/Abstract])) AND (*sugar*[Title/Abstract] OR *ssb*[Title/Abstract] OR 'free sugar'[Title/Abstract] OR 'added sugar'[Title/Abstract] OR 'sugar-sweetened beverages'[Title/Abstract] OR 'cariogenic diet'[Title/Abstract] OR 'dietary sucrose'[Title/Abstract] OR 'dietary sugars'[Title/Abstract] OR 'high fructose corn syrup'[Title/Abstract] OR 'dietary carbohydrates'[Title/Abstract] OR 'carbonated beverages'[Title/Abstract]) | 22,836 |
| 6 | ((*promot*[Title/Abstract] OR *guide*[Title/Abstract] OR *advise*[Title/Abstract] OR *advice*[Title/Abstract] OR *educat*[Title/Abstract] OR *motivat*[Title/Abstract] OR *counsel*[Title/Abstract] OR *info*[Title/Abstract] OR *analysis*[Title/Abstract] OR *train*[Title/Abstract] OR *demonstrat*[Title/Abstract] OR *instruct*[Title/Abstract] OR *behavio*[Title/Abstract] OR *modif*[Title/Abstract] OR *attitude*[Title/Abstract] OR *service*[Title/Abstract]) AND (*health*[Title/Abstract] OR *oral*[Title/Abstract] OR *dental*[Title/Abstract] OR *nutri*[Title/Abstract] OR *diet*[Title/Abstract])) AND (*sugar*[Title/Abstract] OR *ssb*[Title/Abstract] OR 'free sugar'[Title/Abstract] OR 'added sugar'[Title/Abstract] OR 'sugar-sweetened beverages'[Title/Abstract] OR 'cariogenic diet'[Title/Abstract] OR 'dietary sucrose'[Title/Abstract] OR 'dietary sugars'[Title/Abstract] OR 'high fructose corn syrup'[Title/Abstract] OR 'dietary carbohydrates'[Title/Abstract] OR 'carbonated beverages'[Title/Abstract]) | 20,357 |
| 7 | ((*promot*[Title/Abstract] OR *guide*[Title/Abstract] OR *advise*[Title/Abstract] OR *advice*[Title/Abstract] OR *educat*[Title/Abstract] OR *motivat*[Title/Abstract] OR *counsel*[Title/Abstract] OR *info*[Title/Abstract] OR *analysis*[Title/Abstract] OR *train*[Title/Abstract] OR *demonstrat*[Title/Abstract] OR *instruct*[Title/Abstract] OR *behavio*[Title/Abstract] OR *modif*[Title/Abstract] OR *attitude*[Title/Abstract] OR *service*[Title/Abstract]) AND (*health*[Title/Abstract] OR *oral*[Title/Abstract] OR *dental*[Title/Abstract] OR *nutri*[Title/Abstract] OR *diet*[Title/Abstract])) AND (*sugar*[Title/Abstract] OR *ssb*[Title/Abstract] OR 'free sugar'[Title/Abstract] OR 'added sugar'[Title/Abstract] OR 'sugar-sweetened beverages'[Title/Abstract] OR 'cariogenic diet'[Title/Abstract] OR 'dietary sucrose'[Title/Abstract] OR 'dietary sugars'[Title/Abstract] OR 'high fructose corn syrup'[Title/Abstract] OR 'dietary carbohydrates'[Title/Abstract] OR 'carbonated beverages'[Title/Abstract]) | 19,690 |
| 8 | child*[Title/Abstract] | 1,016,645 |
| 9 | (((*promot*[Title/Abstract] OR *guide*[Title/Abstract] OR *advise*[Title/Abstract] OR *advice*[Title/Abstract] OR *educat*[Title/Abstract] OR *motivat*[Title/Abstract] OR *counsel*[Title/Abstract] OR *info*[Title/Abstract] OR *analysis*[Title/Abstract] OR *train*[Title/Abstract] OR *demonstrat*[Title/Abstract] OR *instruct*[Title/Abstract] OR *behavio*[Title/Abstract] OR *modif*[Title/Abstract] OR *attitude*[Title/Abstract] OR *service*[Title/Abstract]) AND (*health*[Title/Abstract] OR *oral*[Title/Abstract] OR *dental*[Title/Abstract] OR *nutri*[Title/Abstract] OR *diet*[Title/Abstract])) AND (*sugar*[Title/Abstract] OR *ssb*[Title/Abstract] OR 'free sugar'[Title/Abstract] OR 'added sugar'[Title/Abstract] OR 'sugar-sweetened beverages'[Title/Abstract] OR 'cariogenic diet'[Title/Abstract] OR 'dietary sucrose'[Title/Abstract] OR 'dietary sugars'[Title/Abstract] OR 'high fructose corn syrup'[Title/Abstract] OR 'dietary carbohydrates'[Title/Abstract] OR 'carbonated beverages'[Title/Abstract]) AND ((2000/1/1:2022/11/3[pdat]) AND (english[Filter]))) NOT (child*[Title/Abstract] AND ((2000/1/1:2022/11/3[pdat]) AND (english[Filter]))) | 16,039 |
| 10 | adult*[Title/Abstract] | 1,138,462 |
| 11 | ((((*promot*[Title/Abstract] OR *guide*[Title/Abstract] OR *advise*[Title/Abstract] OR *advice*[Title/Abstract] OR *educat*[Title/Abstract] OR *motivat*[Title/Abstract] OR *counsel*[Title/Abstract] OR *info*[Title/Abstract] OR *analysis*[Title/Abstract] OR *train*[Title/Abstract] OR *demonstrat*[Title/Abstract] OR *instruct*[Title/Abstract] OR *behavio*[Title/Abstract] OR *modif*[Title/Abstract] OR *attitude*[Title/Abstract] OR *service*[Title/Abstract]) AND (*health*[Title/Abstract] OR *oral*[Title/Abstract] OR *dental*[Title/Abstract] OR *nutri*[Title/Abstract] OR *diet*[Title/Abstract])) AND (*sugar*[Title/Abstract] OR *ssb*[Title/Abstract] OR 'free sugar'[Title/Abstract] OR 'added sugar'[Title/Abstract] OR 'sugar-sweetened beverages'[Title/Abstract] OR 'cariogenic diet'[Title/Abstract] OR 'dietary sucrose'[Title/Abstract] OR 'dietary sugars'[Title/Abstract] OR 'high fructose corn syrup'[Title/Abstract] OR 'dietary carbohydrates'[Title/Abstract] OR 'carbonated beverages'[Title/Abstract]) AND ((2000/1/1:2022/11/3[pdat]) AND (english[Filter]))) NOT (child*[Title/Abstract] AND ((2000/1/1:2022/11/3[pdat]) AND (english[Filter]))) AND ((2000/1/1:2022/11/3[pdat]) AND (english[Filter]))) AND (adult*[Title/Abstract] AND ((2000/1/1:2022/11/3[pdat]) AND (english[Filter]))) | 2,457 |
