# Supplementary material for: Components in downstream health promotions to reduce sugar intake among adults: a systematic review
Source: Nutr J. 2024 Jan 17;23:11. doi: 10.1186/s12937-023-00884-3 (PMC10792802; doi:10.1186/s12937-023-00884-3)
Supplement: Supplementary file 2 — Supplementary Material 2 [file 12937_2023_884_MOESM2_ESM.docx]

Supplementary file 1 of Main keywords in advanced searching

| Main keywords | Enriched keywords |
| --- | --- |
| Adult | **Adult** |
| Health Promotion Intervention | **“Nutrition Policy”**  **“Guidelines as Topic”**  **“Health Education, Dental”**  **“Oral Health/education”**  **“Dental Caries/prevention and control”**  **“Oral Hygiene/education”**  **“Patient Education as Topic”**  **“Health Promotion”**  **“Dental Research”** |
| Sugar Intake | **“Diet”**  **“Diet, Cariogenic”**  **“Dietary Sucrose”**  **“Dietary Sugars”**  **“High Fructose Corn Syrup”**  **“Dietary Carbohydrates”**  **“Diet, Food, and Nutrition”**  **“Sugar-Sweetened Beverages”**  **“Carbonated Beverages”**  **“Sugars”** |
| Behaviour Change | **“health behavior”**  **“health behaviors”**  **“health knowledge, attitudes, practice”**  **“motivation”** |
