# Supplementary material for: Components in downstream health promotions to reduce sugar intake among adults: a systematic review
Source: Nutr J. 2024 Jan 17;23:11. doi: 10.1186/s12937-023-00884-3 (PMC10792802; doi:10.1186/s12937-023-00884-3)
Supplement: Supplementary file 4 — Supplementary Material 4 [file 12937_2023_884_MOESM4_ESM.pdf]

Supplementary File 3 Details of Interventions' Components

Interventions' components	Details of the Components [Articles]
Theoretical or Model Basis	• PRECEDE-PROCEED model, Dick and Carey's Model of Instructional Design [18].<br>• PRECEDE-PROCEED model and Social Ecological Model [23].<br>• PRECEDE-PROCEED and HAPA models [24].<br>• Social Cognitive Theory [36].<br>• Social Cognitive Theory and Social Ecological Model [38].<br>• Social Cognitive Theory framework is supplemented by motivational interviewing (MI) [32].<br>• Social Cognitive Theory and Health Belief Model [30].<br>• Transtheoretical Model [17].<br>• The Transtheoretical Model of Behavioural Change (decisional balance and self-efficacy) and the construct of social support [33].<br>• Behaviour Change Techniques [37].<br>• Behavioural Change Theories [25].<br>• Theory of Planned Behaviour [20].<br>• Motivational Interviewing [29].<br>• Extended Parallel Process Model [31].<br>• "Coventry, Aberdeen & London - Refined" (CALO-RE) taxonomy [21].<br>• Social Ecological and Process-improvement Theories [22].<br>• Social Identity Theory, Cognitive Dissonance Theory, and Social Influence Theory [35].<br>• Self-determination theory, based on increasing individual competency, autonomy, and relatedness [27].<br>• Diabetes Prevention Programme (DPP) and Look AHEAD trials [39].
Providers	• Trained facilitators [16].<br>• Researcher [30].<br>• A commercial provider [17].<br>• Qualified nutritionist, professor of nutrigenomics and nutrigenetics, and doctor [31].<br>• MI coaching by a trained registered dietitian nutritionist [32].<br>• Counselling by trained research staff [33].

	• Health psychologist [21].<br>• A public high school teacher who had previously served as the interventionist [22].<br>• Peer educators [35].<br>• Trained facilitators (university qualified nutritionists and dietitians) [36].<br>• Trained teachers and researchers to deliver the intervention [24].<br>• Coach [37].<br>• Partnership with Grameenphone Bangladesh using a web-based programme capable of sending automated text messages to participants [25].<br>• Junior Public Health Nurses (JPHNS) [26].<br>• Staff in food stores, worksites, schools, and selected media outlets [38].<br>• Clinician and staff [27].<br>• Trained health educator [29].<br>• Postdoctoral level clinicians supervised by a licensed clinical psychologist [39].
When and How Much	**A month**<br>• A daily dose for 28 days [28].<br>• 30-day dietary intervention [37]<br>• 4 computer-tailored newsletters for 4 weeks [34].<br>• 45 minutes sleep consultation session during 1 month [21].<br>**3 months**<br>• Eight weekly and two biweeklies 2.5-hour session for 3 months [16].<br>• Seven dietary and lifestyle counselling hourly session, biweekly for 3 months [30].<br>• 3-month mHealth programme [17].<br>• 60–90 minutes of 9 group sessions delivered weekly for 6 weeks for 3 months [35].<br>**6 months**<br>• 6 months with telephone motivational interviewing up to two calls during months 3 through 5 of the intervention (15–20 minutes length) [33].<br>• 90–120 minutes duration of 3-small-group educational classes at Weeks 1, 6, and 17, one live teach-back call, and 6.9 minutes of 11 interactive voice response telephone call weekly during the first 3 weeks, biweekly for 6 months [20].<br>• 30 minutes duration of six face-to-face education session weekly for 6 months [22].<br>• A theory-based text message a day total up to 90 messages for 6 months [25].

	• 15 minutes MI and a 30-minute video. Five minutes booster telephone call a week, two weeks, and six months post intervention [29].<br>**12 months**<br>• 12 months with 6 different communication sessions [31].<br>**15 months**<br>• 21 mini -educational online lessons, 3 tailored nudges each week plus 1 encouragement to view the lessons; visit the website weekly for 15 months [18].<br>**Others**<br>• Single home visit, fortnightly addition new content in website, text messages and emails weekly [23].<br>• 15-hour guided public health nutrition programme in 6 weeks [36].<br>• 3 times/week harvesting produce, biweekly 1-hour education classes for 4 months [32].<br>• Weekly individualised goal setting, twice per week text messages and optional to access Facebook group [27].<br>• A day training consists of 3 sessions on knowledge (1.5 h), attitude (2 h), and practice/skills (2.5 h) [26].<br>• 6 months [39].<br>• 14 months [38].<br>• 15 months [40].<br>• 24 months [24].
Follow-up Duration	**Less than a month**<br>• A day prior to supplement consumption and day 28 or a close as practical [28].<br>**1-3 months**<br>• 1 month [21, 34, 37].<br>• 1 month and 3 months [16].<br>• 3 months [17, 30].<br>• 6 weeks [36].<br>**3-6 months**<br>• 4 months [27, 32].<br>• Weeks 1, 5, 11, 13 and 17 [39].<br>**6-12 months**<br>• 3 months and 6 months [22].<br>• 6 months [20, 23, 25, 26, 33, 35]<br>• 6 months and 10 months [29].

	**12 months and over**<br>• 12 months [24, 38].<br>• 10 weeks, 6 months, 12 months [31].<br>• 10 weeks, 15 months [18].<br>• 9 months, 15 months [40].
Materials/ Equipment	• CD-ROMs for home use to guide meditation practice 6 days/week [16].<br>• A food consumption diary [30].<br>• Booklet, 192 SMS text, e-mail messages, 4 smartphones applications, internet forums [17].<br>• Lecture on healthy lifestyle and diet, gene-diet interaction, mail of personal apoE genotype [31].<br>• Websites consist of mini-educational lessons and e-mail messages (called nudges) [18].<br>• Telephone counselling, website, mobile phone app, and text messages [19].<br>• Web-portal, the garden [32].<br>• Binders consisting of the nine educational lessons. A Delta Body and Soul cookbook and monthly newsletter featured nutrition and physical activity [33].<br>• Newsletter, Fruit/Vegetables Snacks provided, Pedometers (for physical activity) [34].<br>• A personalised sleep diary [21].<br>• Modules [35].<br>• “SMART Eating” kit– kitchen calendar, dining table mat, and measuring spoons; and “SMART Eating” website [23].<br>• Ozharvest’s Everyday (photo-based) Cookbook [36].<br>• Simple, easy-to-read, and culturally adapted newsletters [24].<br>• Help sheet which details a range of barriers and potential solutions [37].<br>• Training module [26].<br>• Educational posters, newsletter, brochures, flyers, and educational displays [38].<br>• Fitness tracker and a diet tracking app and access to private Facebook group [27].<br>• Food diaries, GOS/placebo supplement, and stool sampling kit [28].<br>• Ecological momentary assessment (EMA) survey to be completed by participants [39].<br>• Health education intervention package on lifestyle modification for adults with metabolic syndrome (MetS) [40].
	• Based on the personal goals [30].

Tailoring	• Tailored to the process of change identified [17].<br>• Tailored messages based on genotype analysis [31].<br>• A personalised website [18].<br>• Tailored to health-related beliefs, barriers, and sociocultural norms [19].<br>• Tailored educational newsletter based on baseline self-reports [34].<br>• Personalised sleep consultation session [21].<br>• Personalised email feedback on the participants' action and coping plans [37].<br>• Individualised goal-setting and tailored advice from clinicians [27].
Delivery Mechanism	**Technology-mediators**<br>• Technology-mediator (SMS text and e-mail messages, smartphone applications, and internet forums) [17].<br>• Technology mediators (internet and e-mail) [18].<br>• Technology mediators (Telephone counselling and website and mobile phone app use, text messages) [32].<br>**Face-to-face**<br>• Face-to-face [26, 28, 29, 31, 35, 36, 40].<br>• Face-to-face in small groups of five [30].<br>• Face-to-face group session [19, 22].<br>• Community-driven approach: Food stores, worksites, and schools [38].<br>• Technology mediators (diet tracking app: Healthwatch360) and social media peer support [27].<br>**Combination**<br>• Face-to face and Technology mediated [16, 21, 33].<br>• Face-to-face and technology mediated (telephone) [34].<br>• Face-to-face and technology mediators (computer) [20].<br>• Face-to-face and technology mediated (internet-delivered) [37].<br>• Face-to-face and technology mediated (smartphone application) [39].<br>• Technology mediated and face-to-face assessment [25].<br>• Face-to-face and technology mediated communication (e.g., short message service or SMS, email, social networking app and "SMART Eating" website) [23].<br>• Face-to-face lifestyle counselling sessions during (first year), personalised SMS messages (second year) [24].
	**Food Frequency Questionnaire (FFQ)**<br>• FFQ [24, 26, 27, 36, 38, 40]; The 158-item Delta FFQ [33].

Assessment Tools	• FFQ adapted for sugar consumption in the local context [37].<br>• A single-item question added in a FFQ known as the Dietary Questionnaire for Epidemiological Studies version 2 (DQESv2) to determine consumption of sugar-sweetened beverages [19].<br>• The valid 110-item Block 2005 FFQ (nutrition quest) [16].<br>• Indian Migration Study FFQ [25].<br>**Food Diaries**<br>• Food diaries [28].<br>• 7-day food diary [21].<br>**Dietary Recalls**<br>• 24-h dietary recalls [22, 34, 39]<br>• 24-h dietary recalls (one weekend and two weekdays) [20].<br>• A 3-day, 24-h recall (two consecutive weekdays and 1 weekend day) [30].<br>**Others**<br>• A 15-item version of a beverage intake questionnaire [29].<br>• National Health Interview Survey (NHIS) 5-factor dietary screener by NHIS [23].<br>• Validated, 26-item Dietary Screener Questionnaire [32].<br>• Online short questions to assess the usual weekly intake of SSB [17].<br>• Questionnaires to measure consumption of foods containing excessive fat and sugar [31].<br>• 8-item questionnaire on how often in the past and in what amounts college students consumed soft, fruit drinks, non-diet energy drinks, and sugar-sweetened specialty coffee drinks [18].<br>• Interview [35].
